# Grip Strength and Sports Performance in Competitive Master Weightlifters

**DOI:** 10.3390/ijerph20032033

**Published:** 2023-01-22

**Authors:** Marianne Huebner, Bryan Riemann, Andrew Hatchett

**Affiliations:** 1 Department of Statistics and Probability, Michigan State University, East Lansing, MI 48824, USA; 2 Department of Kinesiology, Michigan State University, East Lansing, MI 48824, USA; 3 Department of Health Sciences and Kinesiology, Armstrong Campus-Georgia Southern University, Savannah, GA 31419, USA; 4 Department of Exercise and Sport Science, Aiken-University of South Carolina, Aiken, SC 29801, USA

**Keywords:** aging, athletes, snatch, clean and jerk, muscle strength, hand symmetry, World Master Championships

## Abstract

Grip strength (GS) is correlated with major muscle group strength; weakness and asymmetry in older adults are predictive of future disease and functional limitation risk. GS at different ages and hand symmetry for Olympic-style weightlifters and their association with performance have not been established. GS was measured in 164 athletes participating in the 2022 World Master Weightlifting Championships. The objectives wereto study the magnitude of the age-associated decline in GS in weightlifters and the association of GS with weightlifting performance. Hand symmetry was considered as a potential factor in successful lifts. Ages ranged from 35 to 90 (mean 53 years). Participants reported weekly training averages of 8.3 h of weightlifting and 4.1 additional hours of physical activities. The age-associated decline in GS was less steep than the decline in weightlifting performance. GS was lower in weightlifters compared to athletes in other sports that require grasping or force application (t = −2.53, *p*=0.053 for females; t = −2.62, *p*= 0.029 for males). The rate of decline was similar across different populations (weightlifters, other athletes, community-dwelling adults). Height and age were associated with GS, but performance level and training hours were not. GS was associated with snatch performance (t = 3.56, *p* < 0.001) but not with clean and jerk (t = 0.48, *p* = 0.633).

## 1. Introduction

Olympic-style weightlifting requires technical skills, speed, balance, coordination, and strength. Weightlifting consists of two competitive lifts: the snatch, which requires the barbell be lifted from the floor to overhead in one continuous movement, and the clean and jerk, where the barbell is raised to the shoulders and then to an overhead locked out position [1]. Muscles exert maximal forces in a fraction of a second, and the power output in weightlifters exceeds that of other strength athletes such as bodybuilders and powerlifters [2]. The age of peak performance in weightlifting occurs in the mid-twenties, at similar ages for males and females [3,4]. Age-associated performance decline in weightlifting has been studied, where the decline for female weightlifters mirrors the decline for men, except for an accelerated decline during a 10-year period across the age range from the late forties to late fifties coinciding with the transition to menopause [5]. Other sports also requiring power, such as throwing disciplines and high jump in track and field, had a more rapid age-associated decline in their world records compared to those of running disciplines in track and field [6]. The rate of decline was less in strength sports such as powerlifting compared to weightlifting, based on American and world records [7]. Power is the product of force and velocity, whereas strength is based solely upon force; thus, it is not surprising that power sport performance is affected more than strength sport performance given the independent declines in force and velocity that occur with aging [8]. Despite the strength gap in males and females, females may be more likely to preserve their muscles strength than males with increasing age [9,10,11]. A comparison of the rate of decline in overall muscle strength and the rate of decline in power has not been completed in the same athlete population. Master weightlifters are an excellent study cohort for such a comparison because of the age range of the participants and the strength- and power-oriented nature of the sport.

Grip strength (GS) is an indicator of overall strength that has been correlated with strength in major muscle groups [12,13]. Since GS is considered a biomarker for morbidity, and mortality GS decline was studied in community-dwelling adults, leading to the development of normative values [14]. Additionally, considering GS asymmetry in conjunction with GS weakness appeared to improve the value of GS testing in predicting future disease risk and functional limitations in older adults [13,15,16,17]. GS was also evaluated as a potential link with sport performances in several disciplines such as racket sports or ball sports [18] and in weightlifting [19] for youth and seniors in their mid-twenties. Some sports such as baseball, golf, climbing, swimming, water polo, wrestling, judo, handball, and tennis have had more research attention on GS and performance than other sports, including strength sports [18]. The GS decline was studied in a large European population, ages 50 and older, engaged in vigorous physical activities [10] and in athletes participating in the USA National Senior Games, ages 50 and older [20]. GS in Master weightlifters, ages 35 and older, and association of GS with weightlifting performance have not been studied. It is not known whether GS in weightlifters is stronger compared that in older athletes requiring grasping (such as racket sports) or other athletes such as runners. Several studies examined the association between GS asymmetry and health outcomes [13,15], but the relationship between GS asymmetry and sport performance in an aging population is unknown. Olympic weightlifting is a sport predicated on strength being distributed throughout the body and then applied to the movement of a barbell. That movement occurs at the junction of the hand(s) and the barbell. Therefore, GS asymmetry may play a large role in competitive Olympic weightlifting and may change over time as athletes age.

The primary aim was to study the magnitude of the age-associated decline in GS in weightlifters as it relates to other populations and training. The secondary aim was to study the association of GS and GS asymmetry with weightlifting performance. We hypothesized that the decline in GS would be less steep than the decline in weightlifting performance and that greater GS is predictive of weightlifting performance. We expected that more weekly training hours would be associated with higher GS.

## 2. Materials and Methods

### 2.1. Study Participants

Male and female athletes competing at the 2022 World Master Weightlifting Championships hosted in Orlando, FL, USA, were eligible for this study. All data were collected on-site during the World Master Championships in December 2022. Athletes were recruited via email through their National Master Chairs, the Master Weightlifting Facebook site, and on-site word-of-mouth at the competition venue. Exclusion criteria were athletes in the adaptive category and missing anthropometric data. Participants provided written informed consent in accordance with the Declaration of Helsinki, and all study documents and procedures were approved by the institutional research ethics committee (Michigan State University: STUDY00007906).

### 2.2. Measurements

GS and anthropomorphic data were collected on-site in the athlete’s lounge that was used by athletes after weigh-in and technical officials during their breaks. A calibrated hydraulic Jamar dynamometer (Performance Health, Warrenville, IL, USA), with its handle in the second or third position, was used. Participants were instructed to squeeze the dynamometer as hard as they could, while standing with their arms by their sides, flexing their elbows to 90° with forearm kept neutral, and counting to three in an audible voice. Three measurements were taken with each hand, alternating dominant and non-dominant hands. The maximum GS (maxGS) overall and for each hand were calculated. The validity of the dynanometer and reliability of these procedures were demonstrated previously [21,22]. Height was measured using a mechanical stadiometer (Detecto, Webb City, MO, USA).

Participants were asked to complete an investigator-generated questionnaire (Qualtrics; Provo, UT, USA) regarding training history, training hours, and physical activities in addition to weightlifting. Athletes were invited via email and social media to complete the survey at any time during the competition. They were asked about completing the survey at the time of the physical assessments, and a tablet with a survey link was available. Those who had not completed the survey were sent email reminders after the physical assessments. Physical activities were defined as “low impact” (e.g., walking, Pilates, yoga, swimming, cycling), “high impact” (running, ball sports, marital arts, functional movements, gymnastics), or “other strength sports” (powerlifting, body building, resistance training, shot put, discus, javelin).

### 2.3. Statistical Analysis

To compare the performance level of weightlifters, the total weight lifted in competitions is adjusted for body mass and age by calculating the Sinclair–Huebner–Meltzer–Faber (SHMF) formula for females and the Sinclair–Meltzer–Faber (SMF) formula for males (IWF Masters calculations https://www.iwfmasters.org/calculator.html, accessed on 20 December 2022). The Sinclair formula is a logarithmic regression model fitted to world records and Olympic Games performances to adjust for body mass [23]. Continuous variables were summarized with means, standard deviations, and categorial variables with counts and proportions. A multivariable regression model was used to estimate the association of age, body mass, height, hours of training, and performance, as measured by SMF/SHMF with maxGS. We tested cubic functions for age, body mass, and height and then retained only those functional forms that were statistically significant. The association of maxGS with performance in the snatch and in the clean and jerk was assessed with multivariable regression models adjusted for age and body weight. 

The GS ratio = X_D_/X_ND_ was calculated to investigate asymmetry between the hands defined as GS ratio larger than 1.1 or smaller than 0.9 [16]. X_D_ and X_ND_ are the maximum GS measurements for the dominant and nondominant hand, respectively. A Bland–Altman plot was constructed to visualize the agreement between the GS measurements of the two hands. Due to the skewed distribution of the GS ratio, quantile regression for the median using the bootstrap method for the standard error [24] was used to test whether sex, age, body mass index, performance level, or chronic health conditions were associated with the GS ratio.

GS measurements in Master weightlifters at different ages were compared to those of Senior Games athletes who train several hours each week on average, including strength training [20]. Normative GS values from the NIH Toolbox project were published for 5-year age groups and were used as comparison to represent community-dwelling adults [14]. Multivariable regression models were used to compare grip strength in 5-year age categories, including an interaction term between age category and sex. 

All multivariable regression models were evaluated for appropriateness of model assumptions (normal distribution of residuals and checking of residual plots). All analyses were conducted with the statistical software R (version 4.0.3). *p*-values less than 0.05 were considered statistically significant.

## 3. Results

Of the 820 eligible athletes, 172 participated from 17 countries in the physical assessment of the study (21.0%). Four participants with missing anthropomorphic data were excluded. The analysis dataset included 104 females and 64 males, ages 35 to 70 (females) and 35 to 90 (males). There was no statistically significant difference in competition performance (SMF/SHMF) for those who participated and those who did not, but participants were older, because most data collection took place in the first 7 days of the 10-day championships (while the youngest age categories, 35 and 40, were scheduled to compete in the last few days). Of the male participants, 35.5% were 35–49 years, 37.5% were 50–64 years, 15.6% were 65–74 years, 6.3% were 75–84 years, and 3.1% were 85 and older. Of the female participants, 46.2% were 35–49 years, 39.4% were 50–64 years, 13.5% were 65–74 years, and 1.0% was 85–74 years. Descriptive summaries are listed in Table 1.

**Figure 1:**
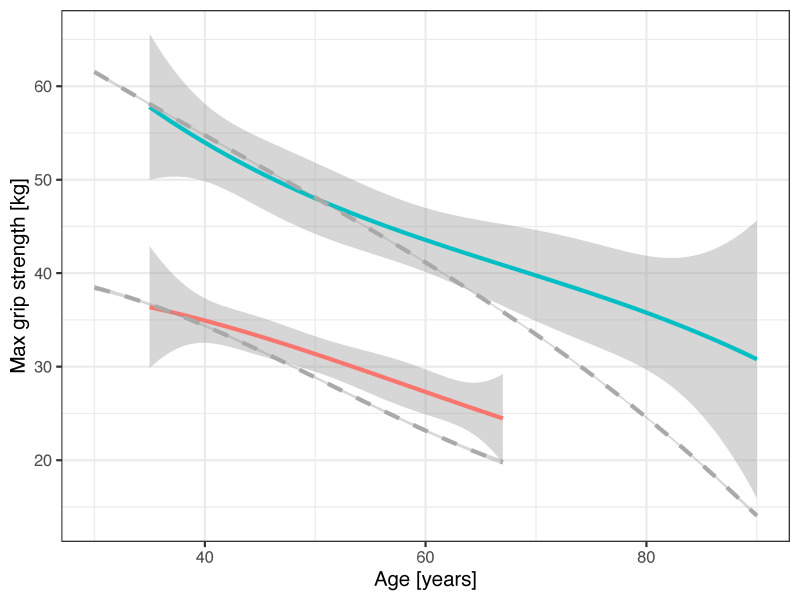
Age-associated decline in GS and weightlifting performance (adjusted for body mass). Figure legend: females—red; males—green. Dashed lines are the dimensionless age factors for performance decline in weightlifting from SMF/SHMF, adjusted to have a comparable intercept with the GS curves.

The age-associated decline in GS is less steep than the corresponding decline in weightlifting, as indicated by the dashed lines derived from the Meltzer–Faber (MF) and Huebner–Meltzer–Faber (HMF) age factors (Figure 1).

**Figure 2:**
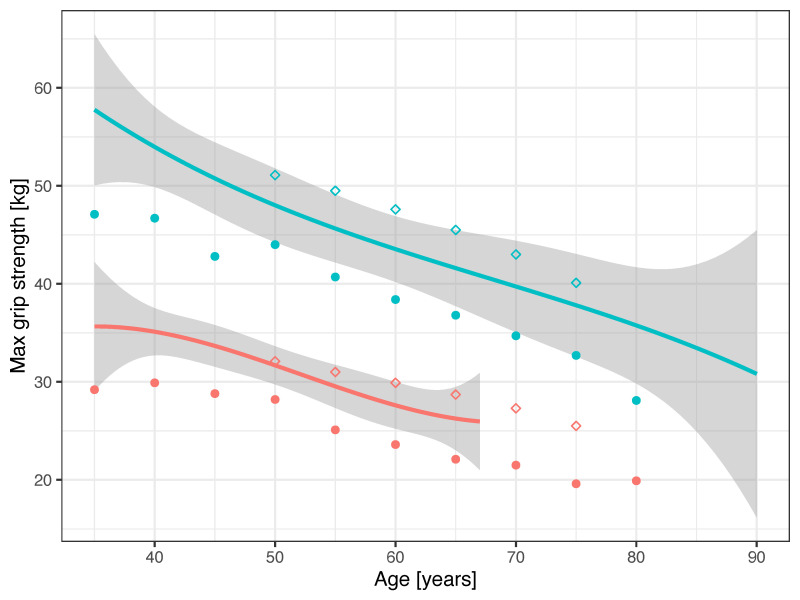
Age-associated decline in maximum GS for females (red) and males (green) with 95% confidence bands. Figure legend: weightlifters—solid lines; community-dwelling adults—closed circles; Senior Games athletes—open diamonds.

GS measurements were higher on average in weightlifters compared to community-dwelling adults [14] across all ages, but they were not statistically significant for males (t = 3.21, *p* = 0.006 for females; t = 0.84, *p* = 0.413 for males) (Figure 2). The GS measurements were lower in weightlifters compared to Senior Games athletes across all ages (t = −2.53, *p* = 0.053 for females; t = −2.62, *p* = 0.029 for males) [20]. Events represented at the National Senior Games include sports where grasping or force application is important, such as racket sports or ball sports [18,20]. The decline with increasing age was similar across the populations, between weightlifters and community dwelling adults (t = −1.36, *p* = 0.196 for females; t = 0.61, *p* = 0.550 for males) and between weightlifters and Senior Games athletes (t = 0.89, *p* = 0.401 for females; t = 2.07, *p* = 0.065 for males).

Better GS was associated with height and younger ages but not with weekly hours of weightlifting training or with better weightlifting performance (Table 2). Body mass was not associated with GS and was not included in the model.

**Figure 3:**
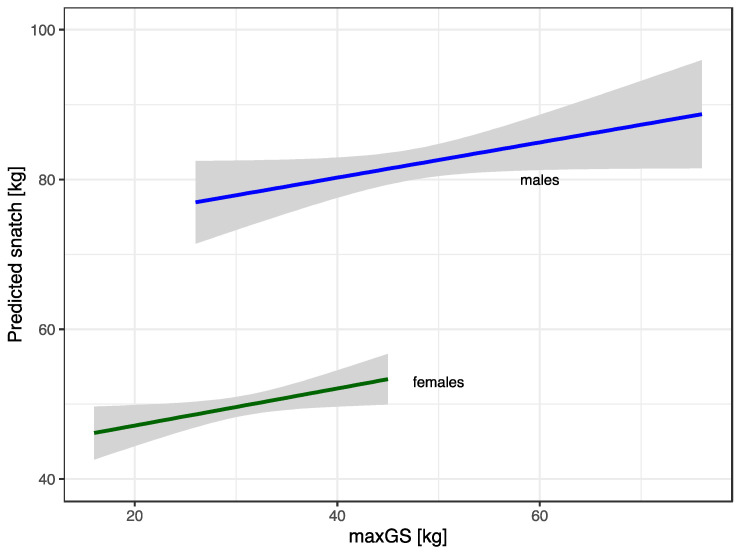
Predicted snatch performance from maxGS with 95% confidence intervals.

Better GS was associated with snatch performance, after adjusting for age, body mass, and weekly training hours (overall t = 3.56, *p* < 0.001), but not with clean and jerk performance (Table 3). The estimated model coefficients associated with snatch performance were similar for females and males, 0.25 ± 0.11 and 0.25 ± 0.11, respectively. Cohen’s f^2^ effect size [25] for maxGS was small: for the models with snatch it was 0.05 for females and 0.07 for males, respectively. The predicted increase in snatch for each additional kilogram in maxGS is shown in Figure 3, corresponding to athletes of average age, height, and body mass, training 8 h each week.

**Figure 4:**
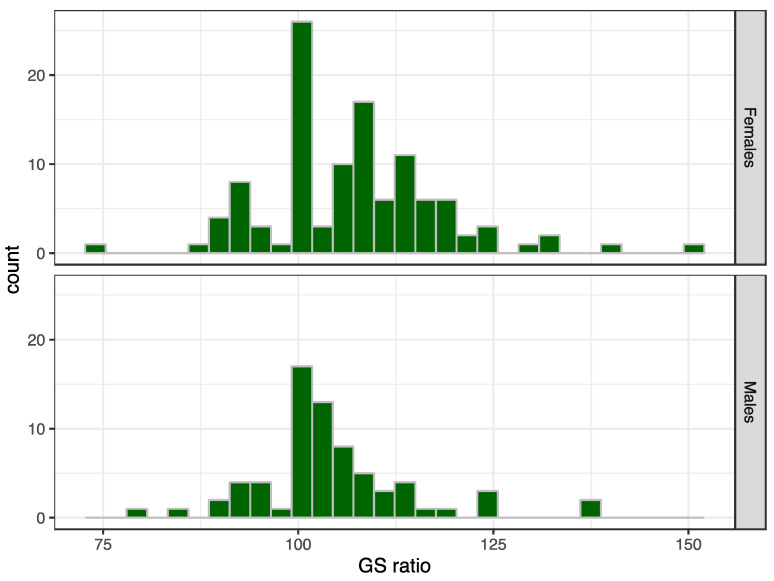
GS ratio for male and female weightlifters.

**Figure 5:**
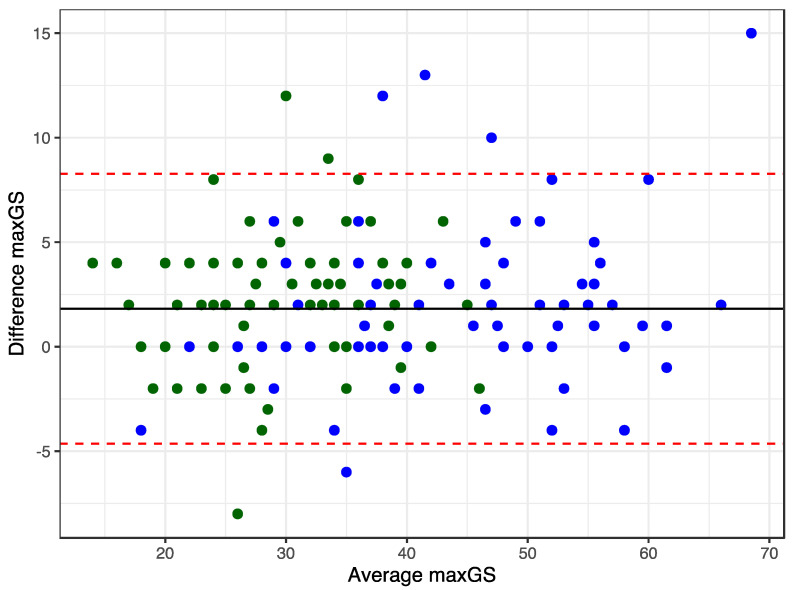
Bland–Altman plot for GS measurements for both hands. Females (green) and males (blue).

The GS ratio was statistically significant different from 1.0, with a skewed distribution toward the dominant hand (*p* < 0.001 for males and for females) (Figure 4). The mean difference between the maxGS of the dominant and non-dominant hands was 1.8 kg. Females had more asymmetry than males (t= −2.29, *p* = 0.023). Hand asymmetry occurred in 30.9% (95% confidence interval: 23.9%, 38.0%) of the weightlifters with a GS ratio larger than 1.1 or smaller than 0.9. There were no chronic medical conditions or prior hand/wrist injuries that could explain the asymmetry for weightlifters falling outside the confidence limits in the Bland–Altman plot (Figure 5). However, two of the eight athletes did not total in the competition, while 7.1% of all competitors did not total. “Not total” means they missed all three lifts of the snatch or of the clean and jerk. Two men had clinically relevant weak GS, defined as less than 26 kg for men and less than 16 kg for women [26]. They were older than 70 years, and one did not total.

## 4. Discussion

The purpose of this investigation was to compare GS decline between weightlifters, community-dwelling adults, and other athletes and to study whether there was a link between GS and weightlifting training habits and performance. The main findings were, first, that the age-associated decline in GS was less steep than the decline in weightlifting performances. Weightlifters had better GS than community-dwelling adults, but worse GS than Senior Games athletes. Second, GS was associated with better snatch performance but not with better clean and jerk performance. Weekly hours of weightlifting training and performance level (SMF/SHMF) were not associated with GS. Third, GS was higher in the dominant hand. Age was not associated with GS asymmetry, and a smaller proportion of weightlifters had GS asymmetry compared to community-dwelling adults.

### 4.1. Age-Associated Decline in GS

GS is considered a reliable measure to assess overall muscle strength and is used in clinical settings for the diagnosis of sarcopenia and frailty across the lifespan. Decreasing muscle quality is associated with age-related decline in GS in middle-aged and older adults [27]. In general, Master athletes are healthier, with a lower prevalence of chronic diseases, compared to sedentary community-dwelling adults. Thus, it is not surprising that the maxGS was higher in weightlifters than that for comparative sex and age groups in community-dwelling adults on average [14]. However, weightlifters presented with lower GS than that of athletes participating in the National Senior Games, for ages 50 years and older. The events at the National Senior Games include sports such as racket sports and ball sports, where grasping or force application is relevant [18,20]. Weightlifters typically use a hookgrip when gripping the barbell, where the fingers are wrapped around the thumb, holding the barbell securely with less muscular effort. The hookgrip may enable athletes to utilize greater loads [28]. In training, athletes may use straps during accessory exercises such as pulls, due to the comfort level. Thus, Olympic-style weightlifting may require lower GS than other sports where gripping is important, for example, archery, badminton, bowling, climbing, racquetball, or tennis. In contrast, the GS force was higher in weightlifters (393 ± 88.3 N) compared to long distance runners, with a similar ratio of males and females (360 ± 59 N) (*p* < 0.001) [29]. In a systematic review, improvements in GS occurred with strength/resistance training interventions, but large effects in favor of the intervention groups were seen in studies with multimodal interventions, where GS benefitted from incorporating strength, balance, flexibility, and endurance in training [30]. Strength, balance, and flexibility are part of weightlifting, but endurance and aerobic exercises would require concurrent training. While most participants in our study reported that they were concurrently training, it is difficult to characterize the modality of these activities, given that we did not ask for those details. Further investigation of GS in weightlifters and comparisons of training diaries would be warranted.

The decline in GS was slower than the decline in weightlifting performance [5]. This was expected: GS is an indicator of overall strength, and weightlifting requires power similar to high jump or throwing disciplines, where a more rapid performance decline is experienced [5,6,7]. Female weightlifters had a slower rate of decline in GS than males. This finding is consistent with other studies, where a slower decline in GS for females compared to males was observed in different populations, such as in a large European cohort engaged in vigorous physical activity at least once a week [31], in a cohort of Senior Games athletes [20], and in community-dwelling adults [14].

### 4.2. GS and Weightlifting Performance

GS was not associated with better overall weightlifting performance as measured by SMF/SHMF. However, GS was associated with better performance in the snatch but not in the clean and jerk. Associations of GS and sport performances were mostly studied in younger athlete populations [18]. To the best of our knowledge, only one study considered GS in junior American weightlifters [19]. The authors observed differences in GS between elite and sub-elite junior Olympic weightlifters, where GS contributed 14% of the explained variance to classify young lifters as elite or non-elite. The snatch is one of the most technical competitions in the sport of weightlifting. One factor that differentiates the snatch from the clean and jerk is that the snatch’s continuous movement aims to lift the bar from the floor to overhead in one motion [1,32]. A successful snatch lift performance is determined by the weightlifter’s ability to lift the barbell overhead and keep it in that position until the confirmation signal sounds [33]. A technical difference between the two competition lifts is the wider hand position of the athlete for the snatch compared to that of the clean and jerk. The snatch requires an overhead squat, where the bar is held in a wide grip overhead in a bottom position. Grip dynamics (position, style, strength) are factors that can impact bar path and athlete position relative to the barbell in all phases of the lift. GS may be a factor that allows the athlete to better control the bar path during the snatch, resulting in a significant relationship between GS and successful snatch performance as well as injury prevention.

Master weightlifters trained an average of 8.4 h of weightlifting, with an additional 4.2 h of other physical activities. Weekly training hours were associated with better performance in the snatch and clean and jerk but were not associated with maxGS. Olympic-style weightlifting is a technical sport. GS is an integral factor in the initial phase of each lift (lifting the bar off the floor). Once the bar begins to move, a transition in significance occurs from grip to technique. Therefore, GS may be a greater factor at the beginning of the lifting motion, while technique (learned movement pattern via training hours) is a greater factor thereafter. Athletes in the Senior Games had higher GS on average, but they trained fewer total hours: 5.6 h of cardiovascular exercise and 1.1 h of strength training each week [20]. Weightlifters typically exceed the strength-based physical activity WHO guidelines [34], but it is not clear whether the WHO guidelines for cardiovascular/endurance activities are met [31]. Weightlifting training sessions vary widely in types of exercises, repetitions, and loads among Master weightlifters due to time constraints, health reasons, and physical capacity. Thus, the correlation of time spent on training and GS may be weak. 

### 4.3. GS Asymmetry

As described previously, evaluating both GS and GS asymmetry increases the prognostic capability of GS testing to indicate future disease risk. Physical activity influences GS asymmetry [35]. Thus, documenting natural bilateral differences in various populations is a necessary. For example, male runners aged ≥50 years demonstrated less GS asymmetry than similarly aged inactive males [36]. In the weightlifters, the GS ratio was 1.06 ± 0.10, which indicated that the dominant side was stronger than the non-dominant side. A similar GS ratio of 1.06 ± 0.15 was found in the National Health and Nutrition Examination Survey (NHANES) [16]. The proportion of weightlifters with GS asymmetry was significantly lower than the corresponding proportion in the NHANES study, which were 30.9% and 41.4%, respectively. GS asymmetry could be due to disuse of the non-dominant hand [16], which is less likely to occur in competitive weightlifters who need to move the bar with both hands. The athletes in this research study train and compete in a sport that requires a relatively high degree of neurophysical engagement. The functional preference for the upper limb influences the occurrence of bilateral differences in other segments of the human body [37]. Neural factors also contribute, in part, to decreased GS during aging [38]. Aging-related shifts in nervous system functioning limit the neural signals for muscle activation that affect strength and muscle mass including GS [13,39]. This is important since individuals with asymmetry or weakness in GS experienced more pronounced limitations in general physical tasks [16]. McGrath and colleagues [13] argued that there is a bidirectional association between GS and neural and motor system functioning. In their study, GS asymmetry was not associated with age or with weightlifting performance level. One of the two male athletes with clinically relevant weak GS did not total in the snatch, and two of the eight athletes with a large GS ratio deviance did not total in the clean and jerk. This was a larger proportion than the 7.1% of athletes overall who did not total in the 2022 World Master Weightlifting Championships. Further studies with more training or competition data are needed to better understand GS and GS asymmetry in weightlifters.

There were some limitations of this study. Our sample included a selection of high-functioning athletes who volunteered for this data collection. However, study volunteers had similar performance levels as measured by SMF/SHMF compared to all other athletes in the competition. Moreover, self-reported training volume could not be verified. As the current study did not include a control or comparison group, reliance upon previously published data was required for interpretation. Thus, the differences identified could be attributable to subtle methodological and protocol differences. Assessment of GS was completed by one of six study team members, which could introduce variability. However, all study team members used a uniform protocol for the assessment, and extensive protocol rehearsal was completed by the team prior to data collection. The previous literature, using a similar dynamometer and methodology, reported strong inter-tester reliability [21,22]. The study is cross-sectional, and, thus, causal relations could not be established. Longitudinal measurements of GS would be desirable, and, because of the portability, short time required, and non-invasiveness of GS testing, it may be feasible to implement hand dynamometer measurements at weightlifting competitions.

## 5. Conclusions

Weightlifters had greater than average GS, but the magnitude was lower than in other sports that require grasping or force application. The age-associated decline in GS as an indicator for overall muscle strength was slower than the decline in weightlifting performance requiring power. GS was associated with snatch performance. Further research is needed to test the association of missed lifts with GS or with GS asymmetry and to examine whether an improvement in GS could mirror an improvement in the snatch. With continued increase in female participation in the sport, the oldest age groups (older than 65 years) could be studied more in-depth.

## Figures and Tables

**Table 1:** Characteristics of the Master weightlifters.

	*n*	Females, *n* = 104	Males, *n* = 64	Overall, *n* = 168
Age, years	168	51.8 ± 9.4	55.4 ± 13.6	53.1 ± 11.3
Age at start of weightlifting, years	163	43.2 ± 11.6	31.4 ± 15.4	38.9 ± 14.2
Height, cm	168	160.8 ± 7.2	171.4 ± 8.9	164.8 ± 9.4
Body mass, kg	168	68.1 ± 16.1	85.0 ± 14.6	74.5 ± 17.6
BMI, kg/m^2^	168	26.1 ± 5.5	28.8 ± 3.5	27.2 ± 5.0
Weekly training, hours	161	8.3 ± 2.7	8.0 ± 2.7	8.2 ± 2.7
Weekly other physical activities, hours	161	4.3 ± 2.6	3.8 ± 2.1	4.1 ± 2.5
PA low impact exercises, %	164	71.0 ^75^⁄_104_	60.0 ^36^⁄_60_	67.7 ^111^⁄_164_
PA high impact exercises, %	164	47.1 ^49^⁄_104_	51.7 ^31^⁄_60_	48.8 ^80^⁄_164_
PA strength exercises, %	164	10.6 ^11^⁄_104_	13.3 ^8^⁄_60_	11.6 ^19⁄^_164_
SMF/SHMF	168	197.1 ± 44.1	276.2 ± 108.6	227.2 ± 84.5
MaxGS, kg	168	30.7 ± 7.1	46.3 ± 11.5	37.02 ± 11.64
MaxGS, newton	168	301.2 ± 69.6	453.9 ± 112.5	359.3 ± 115.3
GS ratio	168	1.07 ± 0.10	1.04 ± 0.10	1.06 ± 0.10
Hand/wrist injury in the last 12 months, %	146	13.8 ^13^⁄_94_	17.3 ^9^⁄_52_	15.1 ^22^⁄_146_

Abbreviations: BMI—body mass index; WHF—waist-to-hip ratio; PA—physical activities; GS—grip strength; SMF—Sinclair–Meltzer–Faber performance adjustment; SHMF—Sinclair–Huebner–Meltzer–Faber performance adjustment; N—number of non-missing values for the variables.

**Table 2:** Predictors of grip strength.

	Females, *n* = 103		Males, *n* = 58	
	coefficient (se)	*p*-value	coefficient (se)	*p*-value
Intercept	−34.61 (13.47)	0.012	−28.22 (26.64)	0.293
Height	0.47 (0.06)	<0.001	0.50 (0.14)	<0.001
Age	−0.29 (0.06)	<0.001	−0.30 (0.09)	0.003
Hours training	0.26 (0.20)	0.207	0.17 (0.47)	0.723
SMF/SHMF	0.02 (0.01)	0.167	0.06 (0.01)	0.120

Abbreviations: se—standard error; SMF/SHMF—Sinclair–Meltzer–Faber/Sinclair–Huebner–Meltzer–Faber adjusted weightlifting totals.

**Table 3:** Association of weightlifting performance with grip strength.

	Females, *n* = 101		Males, *n* = 56	
**Snatch**	coefficient (se)	*p*-value	coefficient (se)	*p*-value
Intercept	78.71 (6.84)	<0.001	91.54 (10.67)	<0.001
MaxGS	0.25 (0.11)	0.028	0.23 (0.12)	0.056
Age	−1.01 (0.08)	<0.001	−1.27 (0.09)	<0.001
Body mass	0.14 (0.04)	0.002	0.343 (0.08)	<0.001
Weekly training hours	0.75 (0.26)	0.004	1.74 (0.40)	<0.001
**Clean and jerk**				
Intercept	101.29 (7.81)	<0.001	148.85 (14.69)	<0.001
MaxGS	0.18 (0.13)	0.155	−0.25 (0.17)	0.157
Age	−1.23 (0.09)	<0.001	−1.76 (0.12)	<0.001
Body mass	0.23 (0.05)	<0.001	0.62 (0.10)	<0.001
Weekly training hours	0.56 (0.29)	0.059	1.50 (0.44)	<0.001

## Data Availability

Data is contained in the article.
